# A Case of Vaping TCH Oil Leading to Vaping Associated Pulmonary Injury: Our Approach to Its Diagnosis, Management, and Recommendations

**DOI:** 10.1155/2020/6138083

**Published:** 2020-01-04

**Authors:** Anshika Singh, Qiuxue Tan, Nicole M. Saccone, David H. Lindner

**Affiliations:** NCH Healthcare System, Naples, FL 34102, USA

## Abstract

Vaping's popularity has grown exponentially since its introduction to the US market in 2003. Its use has sky-rocketed since the unveiling of the vaping pods in 2017 which may account for the advent of the vaping related illnesses we are now seeing. Substances such as nicotine solution, tetrahydrocannabinol (THC) oil, cannabidiol (CBD) oil, and butane hash oil (BHC) packaged in cartridges available in various flavors and concentrations are aerosolized by the heating of metal coils in the e-cigarette/vaping devices. Cases from all over the country have recently been coming to light in which vaping has led to severe acute pulmonary disease or vaping-associated-pulmonary-injury (VAPI). A vast majority of the presenting patients in the reported cases have required hospitalization and intensive care, needing supplemental oxygen and even endotracheal intubation and mechanical ventilation. 98% of patients present with respiratory symptoms (dyspnea, hypoxia, chest pain, cough, hemoptysis), 81% of patients have gastrointestinal symptoms (nausea, vomiting, diarrhea, and abdominal pain), and 100% of patients have constitutional symptoms such as fever, chills, and fatigue/malaise on presentation. Although based on history and clinical presentation it is reasonable to have a high suspicion for VAPI, diagnostic workup to rule out alternative underlying causes such as infection, malignancy, or autoimmune process should be performed before establishing the diagnosis. Computed Tomography (CT) scans of the chest have predominantly shown ground-glass opacity in the lungs, often with areas of lobular or subpleural sparing. Although lung biopsies have been performed on a relatively low number of cases, lung injury patterns so far have shown acute fibrinous pneumonitis, diffuse alveolar hemorrhage, or organizing pneumonia, usually bronchiolocentric, and accompanied by bronchiolitis. Treatment plans that have led to clinical improvement in the reported cases center around high-dose systemic steroids, although there are a lack of data regarding the best regimen and the absolute need for corticosteroids. The role of antibiotics appears to be limited once infection has definitively been ruled out. We present the case of a young male who vaped THC oil and developed severe acute pulmonary injury requiring mechanical ventilation and showed a remarkable response to high dose steroid therapy with improvement in clinical symptoms and resolution of diffuse ground glass opacity on repeat HRCT scan.

## 1. Introduction

An electronic cigarette or “e-cigarette” is a battery-powered device that vaporizes (hence the term “vaping”) a liquid solution in order to simulate smoking without burning the tobacco. Since its introduction in the market in 2003, its use and popularity has grown exponentially. When activated, usually by pressing a button, the metal coil in the device heats and vaporizes the liquid nicotine, emitting steam which is orally inhaled. Water vapor mixed with chemical byproducts are then exhaled. Apart from nicotine, other substances such as tetrahydrocannabinol (THC), cannabidiol (CBD), and butane hash oil (BHC) can also be inhaled using an e-cigarette device. There is an increasing concern surrounding the practice of vaping as well as an existing knowledge gap regarding vaping-induced-pulmonary-injury (VAPI), its precise mechanism of pathogenesis, and the best course of management in VAPI cases. Our case aims to contribute to this growing literature to help others further understand this relatively new syndrome.

## 2. Case Presentation

A twenty-year-old male with no significant past medical history presented to our ER with fever, nonbloody vomiting of gastric content, and epigastric abdominal pain. Symptoms began four days prior to this presentation along with worsening dyspnea, a nonproductive cough, and one episode of submassive hemoptysis. He was recently seen one day prior at an urgent care center, during which a 2-view chest X-ray showed left perihilar and right basilar interstitial prominence. At that time, he was assessed to have community-acquired pneumonia and sent home on 250 mg of oral Azithromycin. Despite taking azithromycin for one day, his dyspnea worsened and he began to cough up yellow-brown sputum. He denied any previous similar symptoms and denied previous hospital admission for pneumonia. He denied any recent sick contacts or recent travel. Family history and past surgical history was negative. Social history was significant for marijuana use and occasional drinking with friends. He informed us that he has been vaping THC oil cartridges for 3-4 years. He denied smoking cigarettes or vaping nicotine.

**Figure 1:**
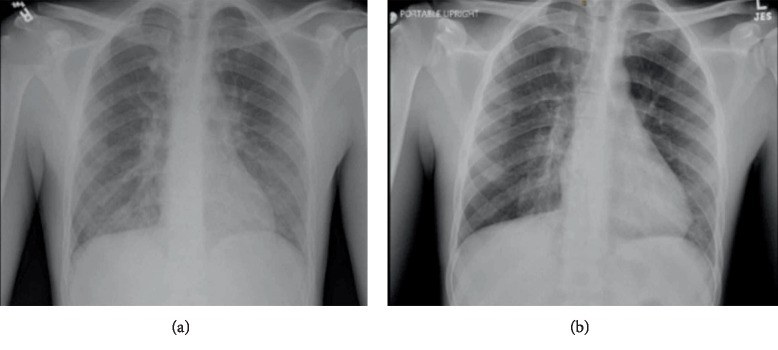
(a) Portable chest X-ray done in the ER on presentation showing bibasilar infiltrates and interstitial prominence. (b) Portable chest X-ray on hospital day 4 prior to discharge with improvement in aeration of the lungs, especially the right lung medially and left lingula. There continues to be minimal interstitial infiltrates present.

**Figure 2:**
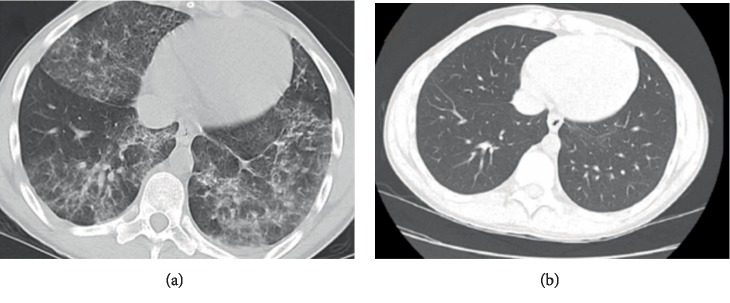
(a) CT scan of the chest without contrast done prior to steroids demonstrating bilateral, multilobar diffuse interstitial and mixed infiltrates evident as “honey combing” and “ground glass” appearance, with well-defined fissure line showing that the infiltrate do no cross between lobes. (b) HRCT scan of the chest post steroid therapy showing resolution of the previously evident diffuse opacity. The scans are 5 weeks apart.

At rest, his respiratory rate was 22/min, heart rate 118/min, blood pressure 135/75 mmHg, temperature 39.3°C, and oxygen saturation 94% on room air. Basic labs were significant for mild leukocytosis of 11.6TH/UL with a neutrophil predominance (92%) and no eosinophils, procalcitonin 0.39, ESR 69, CRP 32, and cannabinoid in urine drug screen. A complete metabolic panel was within normal limits. Blood cultures and respiratory viral PCR were negative. A portable chest X-ray in the ER on this admission showed interval increasing bibasilar infiltrates and persistent left perihilar interstitial prominence (Figure 1), as compared to that from the day prior. Therefore, due to his presenting symptoms, he went for a CT chest which showed bilateral diffuse interstitial and mixed infiltrates in his lungs (Figure 2(a)). Given his acute presentation and suspicion for impending respiratory failure, he was admitted to the critical care unit. Intravenous 125 mg of methylprednisolone was subsequently given, and he was continued on community-acquired pneumonia empiric treatment with ceftriaxone and azithromycin.

However, chest X-rays on the patient's first and second day in the hospital showed no interval improvement of bibasilar infiltrates, and neither did the patient demonstrate any clinical improvement. All the tests ordered to detect infectious and rheumatological causes came back within normal limits (Table 1). Due to the episode of submassive hemoptysis and negative workups thus far, he underwent flexible bronchoscopy on hospital day 2. No biopsies were taken. The serial aliquots did not get bloody, ruling out diffuse alveolar hemorrhage (DAH). Bronchoscopy cultures sent included lower respiratory culture, AFB culture, fungal cultures, viral culture, influenza A/B and CMV, legionella pneumophila PCR, and pneumocystis smear, all of which returned negative. Cytology was negative for malignancy. Flow cytometry results reported lymphocytes: 3%, monocytes: 0%, granulocytes: 95%, CD45 Dim: 0.3%, CD45 Neg: 0.2%. Due to high suspicion for vaping pneumonitis, the patient was given another 120 mg of IV methylprednisolone on hospital day 2.

On hospital day 3, he became increasingly dyspneic, requiring 50 L oxygen via heated high flow nasal cannula. The patient was given another four doses of methylprednisolone 40 mg on this day. On hospital day 4, the patient's dyspnea improved. The chest X-ray taken this day showed improvement in aeration of the lungs, especially the medial right lung and left lingula, along with continued minimal interstitial infiltrates. Antibiotics were discontinued, and he was given another 80 mg of intravenous methylprednisolone prior to hospital discharge on hospital day 4. He was discharged to his home on nine days of tapering prednisone starting at 60 mg daily.

Five weeks after his hospital admission, the patient presented to our pulmonary clinic for a follow-up. He reported feeling “90% back to normal” and said he has been able to help his uncle move some furniture without feeling exhausted or short of breath in doing so. He had felt short of breath with a mild dry cough for the first few days post-hospital discharge; however, he reported that these symptoms have greatly improved. His oxygen saturation was 98% on room air with a respiratory rate of 18/min at rest. His physical exam with respiratory focus was benign. High-resolution chest CT on this day did not convey any findings of acute or active pulmonary parenchymal disease (Figure 2(b)). The previously described bilateral ground-glass opacity and patchy infiltrates encompassing five lobes were no longer present or visible. A pulmonary function test was normal, with no evidence of airway disease.

## 3. Discussion

There is an increasing prevalence of cannabis use in e-cigarettes among the US youth. A study that examined data from about 20,000 middle and high school students regarding the use of e-cigarette devices with a substance other than nicotine reported cannabis use in e-cigarettes by 8.9% of all students and 30.6% of those who ever used e-cigarettes [1]. It is not uncommon for e-cigarette users to view vaping as the healthier alternative to smoking traditional cigarettes. However, in the medical community, there is a growing concern regarding the adverse health implications of vaping, as cases of vaping induced pulmonary injury (VAPI) as seen in our twenty-year-old male patient are being increasingly reported in recent years. So far, about 200 cases have come to light in which pulmonary pathologies such as acute hypersensitivity pneumonitis, lipoid pneumonia, chemical pneumonitis, acute eosinophilic pneumonia, and diffuse alveolar hemorrhage can temporally be related to patients' use of vaping devices. As per the Center for Disease Control and Prevention surveillance case definition, our patient is a “Confirmed” case [2].

The proposed reason explaining why vaping THC oil damages the lung tissue is largely debated and may be the direct impact of cannabinoid vapors or the inflammation from the flavoring and diluting additives in the cartridges. There are growing reports of illegal contamination of TCH cartridges with vitamin-E acetate oil as a diluting agent. Vitamin-E oil, which is otherwise harmless on topical application, is known to cause severe inflammatory reaction in the pulmonary parenchyma when inhaled [3, 4]. Another incriminated agent is diacetyl or 2,3-butanedione, which is a commonly used flavoring agent in the food industry and is now being used as a flavoring agent in vape cartridges to impart a pleasant buttery flavor to the inhaled vapors. The FDA has listed Diacetyl as safe for gastrointestinal consumption, but no regulation exists regarding its use in inhaled tobacco or e-cigarette solutions. Currently, the FDA has not identified any compound present in the cartridges to be the cause of the severe pulmonary injury, although they are working it in collaboration with the CDC while further investigations are in progress. Another potential contributor to pulmonary cytotoxicity is the transfer of metal ions from the heated alloy coils of the devices into the vaping solution and subsequently into the inhaled aerosols [5, 6].

A large case series with a cluster of 53 cases of severe pulmonary disease associated with vaping, reported to the Wisconsin Department of Health Services and the Illinois Department of Public Health, described 98% of patients as having respiratory symptoms (dyspnea, hypoxia, chest pain, cough, and hemoptysis), 81% of patients as having gastrointestinal symptoms (nausea, vomiting, diarrhea and abdominal pain), and 100% of patients as having constitutional symptoms like fever, chills, and fatigue/malaise on presentation. Possibly due to the symptom profile resembling community-acquired pneumonia, patients often end up receiving empiric antibiotics in the ER without much improvement in their condition [7]. Our patient was also prescribed oral azithromycin by the urgent care center to which he had first presented. His condition deteriorated and required him to seek medical attention again after two days. We therefore strongly recommend keeping VAPI high in the differentials, especially in presenting patients who are young and lack any major past medical history that would cause such acute respiratory symptoms. The role of good history-taking skills to guide healthcare practitioners towards this diagnosis cannot be stressed enough. We suggest making it common practice to ask patients if they “vape” instead of limiting the social history to the traditionally-asked question, “do you smoke?” As per a health advisory issued by Pennsylvania Department of Health, information regarding the vaping device used, the substance and the source of the substance, the pattern of vaping, and the timing of use relative to symptom onset must be obtained [8]. A vast majority of the presenting patients in the reported cases have required hospitalization to intensive care, needing supplemental oxygen, and even endotracheal intubation and mechanical ventilation. Two reported cases have also resulted in death due to severe respiratory failure, reemphasizing the need to strongly discourage vaping, regardless of whether it is recreational or an off-label tool to help one quit smoking [7]. Other sources have also reported up to 20–30 deaths in the past year attributable to VAPI [3].

Although based on history and clinical presentation it is reasonable to have a high suspicion for VAPI, a diagnostic workup to rule out infection, malignancy, or autoimmune processes as the underlying cause should be performed before establishing the diagnosis. CT chest scans on several reported cases of VAPI have predominantly shown ground-glass opacity, often with areas of lobular or subpleural sparing. Cases of vaping-associated acute lipoid pneumonia may additionally demonstrate nodular tree-in-bud opacities [7]. Subpleural sparing on chest CT has been noted in some but not all cases and was not present in our case [8]. Despite having mild and nonspecific changes on X-ray, our patient's CT scan showed much more severe interstitial disease and inflammatory changes. Therefore, it would be reasonable to consider obtaining a CT scan of the chest for patients with high suspicion for vaping pneumonitis, regardless of benign-looking chest X-rays. At present, there is no definite guideline established regarding performing bronchoscopy and lung biopsy in either “confirmed” or “probable” cases, but they can be a useful tool to differentiate between various histopathological patterns of VAPI, in addition to ruling out infection, malignancy, and rheumatologic causes of the patients' symptoms as well as excluding diffuse alveolar hemorrhage (DAH). Since this is a newly recognized condition, there are insufficient data regarding the most specific histopathological findings that can be deemed diagnostic of VAPI. A review of lung biopsies from 17 patients with VAPI has shown lung injury patterns ranging from acute fibrinous pneumonitis, diffuse alveolar hemorrhage, or organizing pneumonia, usually bronchiolocentric and accompanied by bronchiolitis [9]. In our patient, a lung biopsy was not obtained; however, negative serial aliquots during bronchoscopy excluded DAH and a cytology report of BAL specimen, which was grossly dark and turbid, showed pulmonary macrophages, neutrophils, and proteinaceous debris, along with pigment-laden histiocytes indicative of a degree of alveolar hemorrhage. No obvious malignant cells or viral inclusions were seen. Flow Differential (%) and Population Analysis reported lymphocytes: 3%, monocytes: 0%, granulocytes: 95%, CD45 Dim: 0.3%, CD45 Neg: 0.2%. His CD4/CD8 ratio of the BAL specimen was 1.38, indicating possible hypersensitivity pneumonitis due to the vaping. Negative antibodies testing for systemic sclerosis, granulomatosis with polyangiitis, SLE, RA factor, AntiCCP, and cryoglobulins helped us rule out the autoimmune conditions from our differentials. We did initially entertain the idea of ANCA negative vasculitis in our patient, however, in absence of renal, upper respiratory tract and cutaneous manifestations it eventually was low on our differentials. We suggest it would be valuable to do BAL with cytology and microbiologic studies and reserve a lung biopsy for specific cases when a better understanding of VAPI's pathogenesis is needed or the possibility of another diagnosis remains. Besides gaining insight about the pattern of pulmonary injury and the extent of damage, it would also help identify other histopathological features that probably have not been recognized or associated with VAPI yet. On our search of the literature, it was interesting to note that there is a knowledge gap regarding correlation between the substance vaped (THC oil vs. butane hash oil vs. various flavored nicotine solutions) and the type of acute lung injury the substance causes.

Treatment plans that have led to clinical improvement in the reported cases center around respiratory support and high-dose systemic steroids, although there is a lack of data regarding the best regimen and the absolute need for corticosteroids. The role of antibiotics appears to be limited once infection has definitively been ruled out. Many centers tend to empirically cover for community-acquired pneumonia. However, clinical improvement has largely been noted, mostly after administering high-dose steroids [7]. Our patient received 120 mg of intravenous methylprednisolone on day 2, day 3, and day 4, followed by 80 mg on day 5 of his hospital stay with the goal of slowly weaning him to oral steroids upon discharge. Again, due to the emerging nature of this syndrome, the total duration of steroid therapy necessary to ensure complete recovery is indeterminate, although tapering the oral dose over 4−6 weeks with repeat lung scans and outpatient follow-up so far appears to be a reasonable course of action. It is still too early to comment on the superiority of one treatment approach over another. High resolution CT (HRCT) scan of the chest in our patient, performed after 5 weeks of initiating steroid therapy, showed complete resolution of the ground-glass opacity and patchy infiltrates in both the right and left lung. A pulmonary function test after the steroid therapy was normal with no evidence of airway disease. Although clinically our patient was back to his baseline, we would like to endorse the importance of regular pulmonary follow-up for these patients in order to watch out for possible long-term complications of vaping THC oil. We did not come across any study looking into the long-term pulmonary outcomes of VAPI following clinical resolution.

## 4. Conclusions

In summary, our case adds to the growing body of literature on VAPI, for which suspicion should be high in patients who use e-cigarettes and present with acute respiratory symptoms. It also goes on to highlight that high dose steroid therapy can be lifesaving. Bilateral ground-glass opacity in the lungs on CT; negative blood, sputum, and BAL culture for infection; and negative rheumatological markers in such a setting are highly suggestive of this diagnosis. The decision to do flexible bronchoscopy with biopsy and BAL specimen cytology should be tailored on a case-by-case basis, but doing so would expand our knowledge of this newly-emerging syndrome.

## Figures and Tables

**Table 1:** Infectious and autoimmune labs tested.

Significant lab	Patient's lab results	Lab reference range
IGE	1900 IU/ml	0–100 IU/ml
IGG	666 mg/dl	700–1600 mg/dl
IGM	143 mg/dl	40–230 mg/dl
IGA	180 mg/dl	70–400 mg/dl
CRP	26.2 mg/dl	<0.3 mg/dl
ESR	69 mm/hr	0–10 mm/hr
Procalcitonin	0.30 ng/ml	<0.05 ng/ml
Lactate dehydrogenase	234 IU/L	87–241 IU/ml
Urine cannabinoids	Positive	Threshold 50 ng/ml
ANA screen	Negative	—
Scl-70	<1.0 AI	—
C3	125 mg/dl	90–180 mg/dl
C4	26.6 mg/dl	10–40 mg/dl
RA	<10 IU/dl	=<15 IU/dl
Cyclic citrullinated peptide	16	Weak positive: 20–39
ANCA	Negative p-anca, c-anca, atypical anca	—
Cryoglobulins	None detected	—
Glom basement Membrane IGG	<1.0 AI	—
AFB culture and smear	Negative	—
Fungus culture and smear	Negative	—
Legionella culture	Negative	—
Pneumocystis smear	Negative	—
Lower respiratory culture	Negative	—
*S. pneumoniae* Antigen urine	Negative	—
Urine culture	Negative	—
Influenza A/B antigen	Negative	—
RP panel	Negative	—
HIV Ab/Ag, 4^th^ gen	Nonreactive	—
